# Under Threat, Weaker Evidence Is Required to Reach Undesirable Conclusions

**DOI:** 10.1523/JNEUROSCI.3194-20.2021

**Published:** 2021-07-28

**Authors:** Laura K. Globig, Kristin Witte, Gloria Feng, Tali Sharot

**Affiliations:** ^1^Affective Brain Lab, Department of Experimental Psychology, University College London, London WC1H 0AP, United Kingdom; ^2^The Max Planck UCL Centre for Computational Psychiatry and Ageing Research, University College London, London WC1H 0AP, United Kingdom; ^3^University of Tübingen, Tübingen 72074, Germany; ^4^The Max Planck Institute for Biological Cybernetics, Tübingen 72076, Germany; ^5^Yale University, New Haven, Connecticut 06520-8205

**Keywords:** anxiety, evidence accumulation, sequential sampling, stress, threat, valence

## Abstract

Critical decisions, such as in domains ranging from medicine to finance, are often made under threatening circumstances that elicit stress and anxiety. The negative effects of such reactions on learning and decision-making have been repeatedly underscored. In contrast, here we show that perceived threat alters the process by which evidence is accumulated in a way that may be adaptive. Participants (*n* = 91) completed a sequential evidence sampling task in which they were incentivized to accurately judge whether they were in a desirable state, which was associated with greater rewards than losses, or an undesirable state, which was associated with greater losses than rewards. Before the task participants in the “threat group” experienced a social-threat manipulation. Results show that perceived threat led to a reduction in the strength of evidence required to reach an undesirable judgment. Computational modeling revealed this was because of an increase in the relative rate by which negative information was accumulated. The effect of the threat manipulation was global, as the alteration to evidence accumulation was observed for information which was not directly related to the cause of the threat. Requiring weaker evidence to reach undesirable conclusions in threatening environments may be adaptive as it can lead to increased precautionary action.

**SIGNIFICANCE STATEMENT** To make good judgments, people gather information. As information is often unlimited, a decision has to be made as to when the data are sufficiently strong to reach a conclusion. Here, we show that this decision is significantly influenced by perceived threat. In particular, under threat, the rate of negative information accumulation increased, such that weaker evidence was required to reach an undesirable conclusion. Such modulation could be adaptive as it can result in enhanced cautious behavior in dangerous environments.

## Introduction

Many important decisions are made when people feel stressed and anxious (Beilock, 2010). Consider a doctor in the operating theater who needs to decide on the best course of action, a soldier on the battlefield who must decide whether to attack, or a driver stuck in traffic selecting which route to take. Whether calm or stressed, to make good decisions people need to gather information over time (Platt and Glimcher, 1999; Usher and McClelland, 2001). For example, a doctor may decide to consult multiple colleagues before deciding to amputate. Because information can be unlimited, an agent needs to determine when the available data are strong enough to make a judgment (Gluth et al., 2012, 2013). Here, we examine how perceived threat impacts the process by which evidence is accumulated to reach a judgment.

A feature of threatening environments is that the potential for adverse outcomes is high. In these instances, it is adaptive to err on the side of caution. For example, imagine you are walking through a dark alley and hear a “pop.” The sound may be a gunshot or perhaps uncorking of a champagne bottle. Interpreting the sound as the former will cause you to escape and mitigate potential risk. Thus, under perceived threat, it may be adaptive to interpret a stimulus as undesirable even if the strength of the evidence supporting such judgment is only limited. The psychophysiological reaction induced by threat can provide a global, rather than specific, danger signal. We thus hypothesized that the effects of threat on evidence accumulation may be observed even when the source of the threat is unrelated to the decision at hand (e.g., a psychophysiological reaction triggered by a professional conflict may impact how the “pop” is interpreted).

Computationally, this process may occur in at least two ways. First, under perceived threat, people may be predisposed toward undesirable judgments before attaining any information (e.g., you may believe the road you are walking down is dangerous before observing any evidence to that effect). A second, not mutually exclusive possibility, is that under perceived threat an undesirable piece of evidence (e.g., an anxious looking man walking down the road) drives beliefs toward an undesirable judgment (“this road is dangerous”), more so than a desirable piece of evidence (e.g., people are walking past you relaxed and happy) toward a desirable judgment (“this road is safe”). These two distinct mechanisms will result in the same observable behavior. In particular, weaker evidence will be needed to support undesirable judgments under perceived threat.

To tease apart these mechanisms, we used a sequential sampling model to model noisy evidence accumulation toward either of two judgment thresholds (Ratcliff, 1978; Ratcliff and Rouder, 1998; Voss et al., 2013). The model allows estimation of both (1) starting point and (2) rate of evidence accumulation, reflecting the quality of information processing. We can then measure whether either of these factors are influenced by the desirability of a judgment and how this is influenced by perceived threat.

We exposed participants to an acute threat manipulation in the lab (Garrett et al., 2018), or a control condition, and then asked them to complete an evidence accumulation task (Gesiarz et al., 2019) that was unrelated to the cause of the threat. In the task, participants witness various stimuli that are contingent on which one of two hidden states they are in. One state was associated with greater rewards than losses (desirable state) and the other with greater losses than rewards (undesirable state). Participants had no control over which state they were in; their task was simply to judge the state, gaining additional rewards for accurate judgments and losing rewards for inaccurate judgments. Thus, it is in participants' interest to be as accurate as possible. They were allowed to accumulate as much evidence as they wished before making a judgment. We examine whether and how perceived threat impacts the accumulation of evidence toward a judgment.

## Materials and Methods

### Experimental design

#### Participants

A total of 91 individuals participated in this study at two sites: University College London (UCL; *N* = 51) and Massachusetts Institute of Technology (MIT; *N* = 40). They were recruited via the participant pools of UCL and MIT. All analyses were repeated separately for participants tested in the two different locations (MIT, UCL). There were no differences between locations in any of our results including model-free analysis, psychometric equations, or DDM analysis.

Participants gave written, informed consent and were remunerated £7.50/$15 for their participation plus an unspecified performance-related bonus. Ethical approval was provided by the Research Ethics Committees at UCL and MIT. One participant who terminated the experiment early and another who failed all comprehension checks were excluded from the analysis. In addition, we followed the exclusion criteria previously published for this task (Gesiarz et al., 2019): we excluded two participants whose accuracy rate was below chance (50%) and four who provided responses based only on the first stimulus in over half the trials. Thus, data of 83 participants was included in the analysis (M_age_ = 30.29, SD_Age_ = 12.20; 37 females, 46 males, 43 at UCL and 40 at MIT). Each participant was randomly assigned to either the threat manipulation group (*N* = 40, M_age_ = 28.98, SD_Age_ = 11; 14 females, 26 males, 21 at UCL and 19 at MIT) or the control group (*N* = 43, M_age_ = 31.51, SD_Age_ = 13.23; 23 females, 20 males, 22 at UCL and 21 at MIT).

#### Manipulation procedure and manipulation check

We followed the exact same threat manipulation as in Garrett et al. (2018). Participants assigned to the threat manipulation group were informed that at the end of the experiment they would be required to deliver a speech on a surprise topic, which would be recorded on video and judged live by a panel of staff members. They were shown an adjacent room where chairs and tables were already organized for the panel. This manipulation is a variation of the Trier social stress test (TSST; Birkett, 2011) with the key difference being that participants in this task were threatened by the possibility of a stressful social event and completed the main task under anticipation of the threat, but the threat was never executed. Having the participants believe the threatening event will take place at the end of the task, rather than before, increased the likelihood that participants' anxiety levels remained high throughout the task. In addition, participants were presented with six difficult mathematical problems that they were asked to try and solve in 30 s. The exact same manipulation procedure previously executed in our lab has been shown to significantly heighten cortisol levels, skin conductance and self-reported state anxiety (Garrett et al., 2018). We have also shown that the manipulation-induced changes in self-reported state anxiety (measured using the Spielberger state trait anxiety inventory; STAI) correlated across participants with physiological indicators of stress (Garrett et al., 2018).

Participants assigned to the control group were informed that at the end of the experiment they would be required to write a short essay on a surprise topic, which would not be judged. They were then presented with six elementary mathematical problems to solve in 30 s. This control manipulation has been shown not to heighten cortisol levels, skin conductance and self-reported state anxiety (Garrett et al., 2018). As a manipulation check, before and after the induction procedure, we asked participants to complete the STAI (Marteau and Bekker, 1992) as a measure of anxiety.

#### Behavioral task

**Figure 1.**
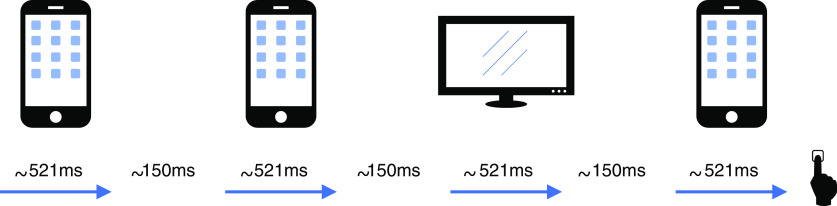
The factory task. In each trial, participants see an animated sequence of televisions and telephones passing along a conveyor belt. Their task is to accurately determine whether they were in a telephone factory, i.e., a factory that produces telephones most of the time, or a television factory, i.e., a factory that produces televisions most of the time. They are incentivized for accuracy and can enter their judgment whenever they like. Each participant is “invested” in one factory. On trials where they happen to be in that (desirable) factory they gain points, on trials in which they happen to be in the other (undesirable) factory they lose points. Notably, this bonus is beyond participants' control and is not affected by the actual judgment made. Stimulus presentation time was jittered, so that participants were less likely to have a clear expectation of when the next stimulus would be observed. Stimulus presentation time on average was ∼521 ms. The lag between stimuli was on average ∼150 ms.

After completing the threat/control manipulation, participants played 80 trials of the “factory game,” published previously by Gesiarz et al. (2019). On each trial, participants witnessed an animated sequence of televisions and telephones passing along a conveyer belt (Fig. 1). There were two types of trials: telephone factory trials and television factory trials. In telephone factory trials, the probability of each item in the animated sequence being a telephone was 0.6 and of being a television 0.4. For television factory trials, the proportions were reversed. The trial type was randomly determined with replacement on every trial with an equal probability for each trial type. Participants were tasked with judging whether they were in a telephone factory trial or a television factory trial. Since the trial type was not directly observable, their means of doing this was through reverse inference over the sequence of objects they were seeing. Participants were free to respond as soon as they wished after initiating the trial and the sequence would continue until they made their choice.

Participants began the game with an endowment of 5000 points. Each 100 points was worth 1 pence/1 cent. One of the two factory types was randomly assigned per participant to be the desirable factory type and the other to be an undesirable type. Participants were informed that each time they visited the desirable factory (desirable state), they would win points, and each time they visited the undesirable factory (undesirable state), they would lose points. We did not specify the exact number of points they would win or lose. Crucially, this bonus was entirely outside of the participants' control, i.e., it was not affected by the judgments the participants made. Separately, participants were informed that they would earn an unspecified number of points for making a correct judgment and lose an unspecified number of points for making an incorrect judgment. We informed subjects that the magnitude of each unspecified bonus/loss were independent of each other, potentially unequal and varied randomly on each trial.

The task was the same as published previously (Gesiarz et al., 2019), except that we jittered the presentation time of the stimuli, so that participants were less likely to have a clear expectation of when the next stimulus would be observed. Because of a technical error this jitter was slightly different across sites (average stimuli presentation time at UCL: 657.28 ms, SD = 1060.73 ms; MIT: 373.65 ms, SD = 49.69 ms). The lag between stimuli was ∼150 ms.

Trials in which participants made their judgment before observing the second object were removed. In cases where a participant did this in over half their trials, we assumed that the participant was not appropriately engaging with the task and eliminated the entirety of their trials. Following Gesiarz et al. (2019), we dropped four participants for this reason, as well as a further 72 responses made before seeing the second item.

#### Training

Before playing the task, participants received extensive instructions and were required to answer multiple-choice comprehension check questions on the key points of the task, with the question repeated until they either chose correctly or failed three times, on which the correct answer was displayed. The comprehension check questions addressed the following key points of how the game worked: that telephone factories mostly produced telephones, but sometimes produced televisions; the bonus for visiting desirable factories was independent of the judgments they made; which factory was their desirable factory; and that trial types (i.e., if they were in a TV or phone factory) were randomly determined and it was not guaranteed that they would see exactly the same amount of each type of factory. Participants then played a practice session of 20 trials, where the trial type was visibly displayed to them (i.e., if they were in a TV or phone factory), so they could have prior experience of the outcome contingencies and the trial type distribution.

### Statistical analysis

#### Manipulation check

An independent two-tailed *t* test was computed to assess the difference in percentage change in STAI [(post-STAI – pre-STAI)/pre STAI] between the threat and control group. One-sample *t* tests were computed to assess percentage change against zero within each group.

#### Psychometric function

We followed the same analysis as in Gesiarz et al. (2019) to relate participants' judgments to the strength of evidence they observed. We fitted a psychometric function, using a generalized mixed effects equivalent of a logistic regression, with fixed and random effects for all independent variables. We fitted these functions separately for participants for whom TV factory was desirable and for whom TV factory was undesirable, and separately for each group (control, threat).

$$P(TV)=\frac{1}{1+e^{-(\beta_{1}X-\beta_{0})}}$$

where P(TV) is the probability of a participant indicating they are in a TV factory; X is the proportion of TV stimuli out of all stimuli observed in a trial. This variable was centered, thus ranging from 0.5 when all samples were TVs to −0.5 when all samples were phones; β_0_ is the indifference point, reflecting the proportion of TVs required to respond TV 50% of the time. If β_0_ = 0, participants would indicate they are in a TV factory half the time when half the samples were TVs. When β_0_ is low the function will move left and vice versa; β_1_ is the slope, reflecting by how much the probability of a participant indicating they are in a TV factory increases when the proportion of TVs increases by one unit.

#### Drift-diffusion modeling

Our aim in modeling our task using the drift-diffusion framework was to assess how perceived threat impacted the evidence accumulation process. In particular, we wanted to assess (1) whether the evidence accumulation process in the threat and control groups was best represented by the same model or a different model; and (2) whether perceived threat impacted the parameters of the evidence accumulation process in our data.

We implemented and compared four different specifications of a DDM (see Table 1). The models included the following parameters: (1) *t*_(0)_, amount of non-accumulation/non-decision time; (2) α, distance between decision thresholds; (3) z, starting point of the accumulation process; and (4) v, drift rate, is the rate of evidence accumulation. Crucially, in models 1 and 3, the starting point was fixed to 0.5, while in models 2 and 4, we allowed the starting point to vary toward one threshold (its value could vary between 0 and 1, thus allowing a valence-dependent starting point bias). In models 1 and 2 with an unbiased drift rate, the parameter was symmetric for desirable and undesirable factories (v and –v). In models 3 and 4, we allowed the drift rate to vary (which we call a valence-dependent drift rate bias) depending on whether the participant was visiting a desirable factory or an undesirable factory (thus allowing a process bias). In these models, we included a term reflecting the difference between drift rates for desirable and undesirable factories (β1 factory desirability). “Factory desirability” is the true factory visited coded as 1 for desirable factories and 0 for undesirable factories. Positive values indicated a bias toward desirable judgements, and negative values indicated a bias toward undesirable judgements. β0 is a constant for the drift rate.

**Table 1.** Drift diffusion model specification

Number	Model	Starting point (z)	Drift rate (5)
1	Valence independent	z = 0.5	v
2	Valence-dependent starting point	0 < z < 1	v
3	Valence-dependent drift rate	z = 0.5	v = β0 + β1 factory desirability
4	Valence-dependent drift rate and starting point	0 < z < 1	v = β0 + β1 factory desirability

For each group, we ran four models which differed in whether we allowed the starting point to vary (models 2 and 4), whether we included a valence-dependent drift rate bias (models 3 and 4), or neither (model 1).

We used the HDDM software toolbox (Wiecki et al., 2013) to estimate the parameters of our models. The HDDM package employs hierarchical Bayesian parameter estimation, using Markov chain Monte Carlo (MCMC) methods to sample the posterior probability density distributions for the estimated parameter values. We estimated both group-level parameters as well as parameters for each individual participant. Parameters for individual participants were assumed to be randomly drawn from a group-level distribution. Participants' parameters both contributed to and were constrained by the estimates of group-level parameters. In fitting the models, we used priors that assigned equal probability to all possible values of the parameters. Models were fit to log-transformed RTs as done previously (Gesiarz et al., 2019), because RTs were non-normally distributed and had a heavy positive skew. Also, since our “error” RT distribution included relatively fast errors we included an intertrial starting point parameter (sz) for both models to improve model fit (Ratcliff and Rouder, 1998). We sampled 20,000 times from the posteriors, discarding the first 5000 as burn in and thinning set at 5. MCMCs are guaranteed to reliably approximate the target posterior density as the number of samples approaches infinity. To test whether the MCMC converged within the allotted time, we used Gelman–Rubin statistic (Rubin and Gelman, 1992) on five chains of our sampling procedure. The Gelman–Rubin diagnostic evaluates MCMC convergence by analyzing the difference between multiple Markov chains. The convergence is assessed by comparing the estimated between-chains and within-chain variances for each model parameter. In each case, the Gelman–Rubin statistic was close to one (<1.1), suggesting that MCMC were able to converge. To assess whether the parameters describing the bias in prior and drift rate are significantly different in the control and threat group, we compared 95% confidence intervals (CIs) of the parameters' values. In addition, model fits were compared using the deviance information criterion (DIC; Spiegelhalter et al., 2002), which is a generalization of the Akaike information criterion (AIC) for hierarchical models. The DIC is commonly used when the posterior distributions of the models have been obtained by MCMC simulation (Gamerman and Lopes, 2006). It allows one to assess the goodness of fit, while penalizing for model complexity.

To validate the winning model, we used each group's parameters obtained from participants' data to simulate log RTs and judgments separately for the threat and control group. We used the exact number of subjects, total number of trials and trial structure as in the experiment. Simulated data were then used to (1) perform model recovery analysis and (2) to compare the pattern of participants' response to the pattern of simulated responses, separately for each group. We sampled 2000 times from the posteriors, discarding the first 500 as burn in. Simulation and model recovery analysis were performed using the HDDM software toolbox (Wiecki et al., 2013).

#### Proportion of correctly identified factories

We computed a linear mixed effects model to assess how group (control/threat) and valence of factory visited (desirable factory/undesirable factory) affected proportion of correctly identified factories as desirable or undesirable. Group, valence of factory, and group × valence of factory interaction were included as fixed and random variables. We included both fixed and random intercepts. We compared the pattern of results obtained from participants' real data to those obtained from the simulated data.

## Results

### Threat manipulation was successful

**Figure 2.**
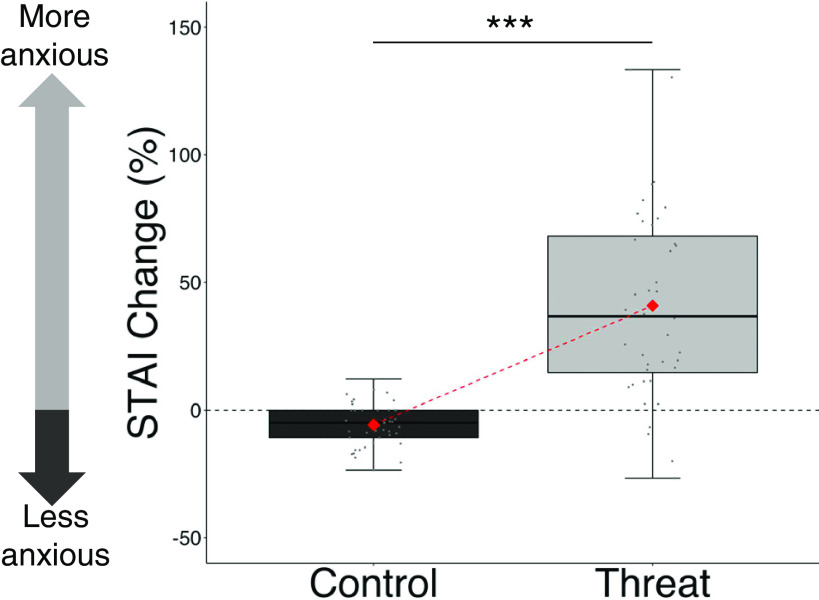
Threat manipulation was successful. Participants in the threat group became significantly more anxious after the manipulation than in the control group. Data are plotted as box plots for each condition, in which horizontal lines indicate median values, boxes indicate 25/75% interquartile range and whiskers indicate 1.5 × interquartile range. Red diamond shape indicates the mean percentage change in STAI per experimental group. Individuals' percentage STAI change are shown separately as gray circles; ***p < 0.001.

The manipulation was successful in inducing perceived threat. Participants in the threat group reported a significantly higher increase in anxiety as a result of the manipulation (increase in STAI score after the manipulation relative to before M = 40.82%, SD = 37.49, *t*_(39)_ = 6.89, *p* < 0.001), compared with those in the control group, who in fact showed a reduction in anxiety (M = −5.74%, SD = 8.24, *t*_(42)_ = −4.57, *p* < 0.001, difference between the two groups: *t*_(82)_ = −7.94, *p* < 0.001, d' = 1.715; Fig. 2) an effect often observed in control participants, who tend to relax as they learn more about the task at hand (Garrett et al., 2018).

### Under threat, participants required weaker evidence to conclude they are in an undesirable factory

We first examined whether perceived threat alters the strength of evidence participants require to make desirable and undesirable judgements. To that end, we fit a psychometric function to the data which relates the percentage of TVs observed on a trial (i.e., the strength of the evidence to judge a factory as TV) to participants' judgment on whether they are visiting a TV or telephone factory. This was done separately for participants for whom the TV factory was desirable and for whom it was undesirable in the threat and control group.

**Figure 3.**
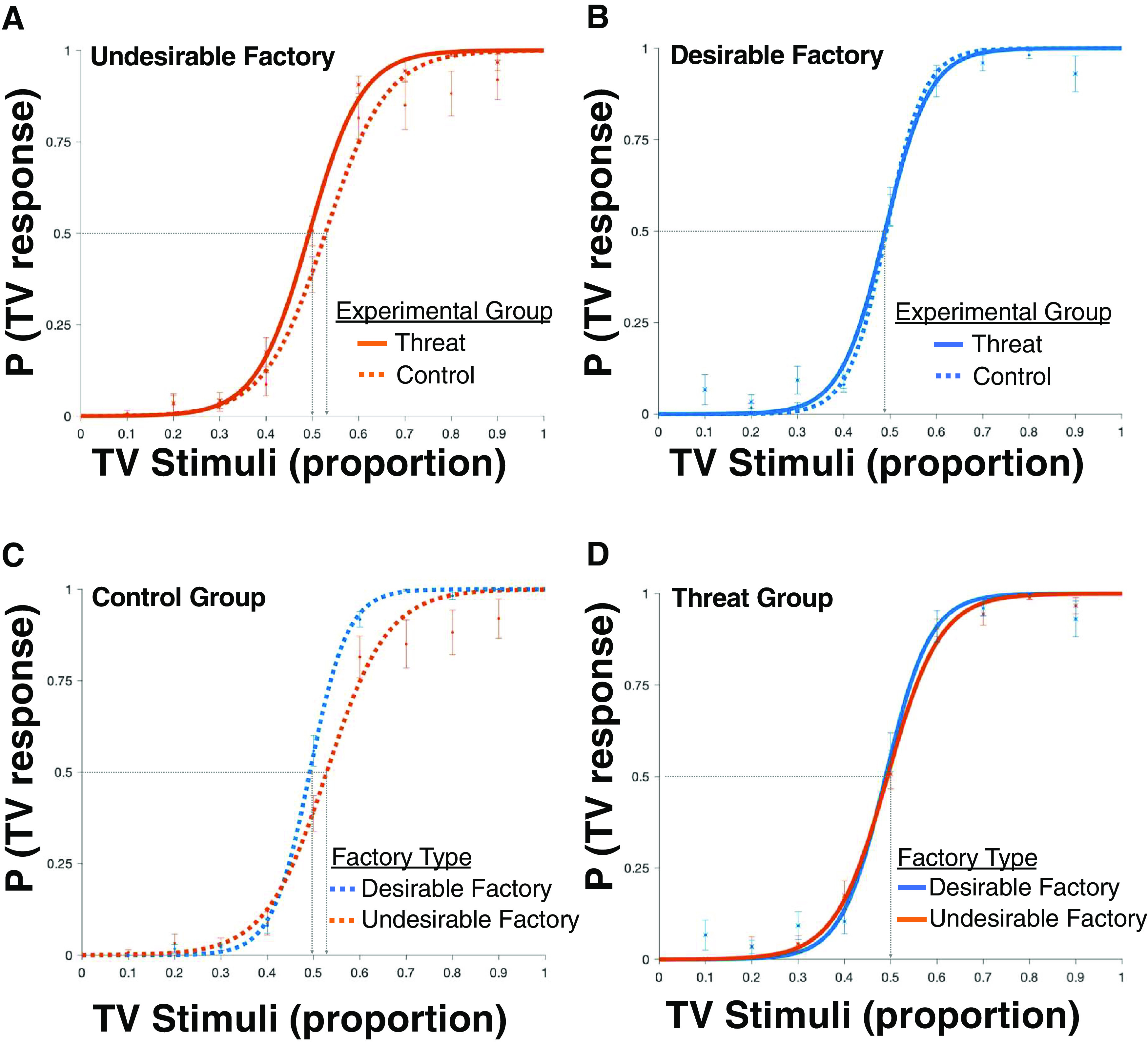
Under threat, weaker evidence is required to reach undesirable conclusions. Fitted psychometric function for data of the threat group (solid line) and control group (dotted line); *y*-axis shows the proportion of times participants indicated they were in a TV factory as a function of the proportion of TV items they observed in a trial before making a judgment (*x*-axis). In blue are the data of participants for whom the TV factory was the desirable factory. In orange is the data of participants for whom the TV factory was the undesirable factory. (***A***) Compared with control, under perceived threat, participants required a smaller proportion of TVs to be observed before reaching the conclusion that they were in a TV factory, when the TV factory was undesirable. This can be seen as the solid line (threat group) is shifted left relative to the dotted line (control group). (***B***) No such difference is observed when the TV factory is desirable. (***C***) Participants in the control group required a smaller proportion of TVs to be observed before reaching the conclusion that they were in a TV factory, when the TV factory was desirable than undesirable. This is seen as the blue line (desirable) is shifted left relative to the orange line (undesirable). (***D***) This difference is abolished under perceived threat. Error bars show standard error of the mean (SEM) at given level of proportion of TVs observed (error bars for threat group are indicated by “x” at the center of the error bar). Gray dashed line indicates point of indifference, i.e., how much evidence is needed for participants to say “TV” half the time.

As observed in Figure 3*A*, under perceived threat, the psychometric function of participants for whom the TV factory was undesirable (solid orange line) was shifted left compared with controls (dotted orange line). This means that compared with control, under threat, participants required a smaller proportion of TVs to be observed before reaching the conclusion that they were in a TV factory when the TV factory was undesirable (indifference parameter was higher for the threat group: β_0_ = 0.11, 95% CI [−0.19, 0.41], than controls: β_0_ = −0.58, 95% CI [−0.96, −0.20], d' = 0.61; Fig. 3*A*). No such difference is observed when the TV factory is desirable; participants in both groups require an equal proportion of TVs to be observed before reaching the conclusion that they were in a TV factory. This can be seen in Figure 3*B* where the psychometric function for threat and control participants overlap (indifference parameter was not different for the threat group: β_0_ = 0.28, 95% CI [−0.07, 0.62] and control group: β_0_ = 0.23, 95% CI [−0.08, 0.53], d' = 0.045; Fig. 3*B*).

While controls required weaker evidence to conclude they were in a desirable factory than undesirable factory (replicating previous findings; Gesiarz et al., 2019), the difference was abolished under perceived threat. This can be observed where the psychometric function of control participants for whom the TV factory was desirable (Fig. 3*B*, dotted blue line) is shifted to the left of control participants for whom the TV factory was undesirable (Fig. 3*C*, dotted orange line; indifference parameter was greater for desirable factory: β_0_ = 0.23, 95% CI [−0.08, 0.53] than undesirable: β_0_ = −0.58, 95% CI [−0.96, −0.20], d' = 0.694; Fig. 3*C*), while for participants in the threat group they overlap (indifference parameter when the TV factory was desirable β_0_ = 0.28, 95% CI [−0.07, 0.62] and undesirable β_0_ = 0.11, 95% CI [−0.19, 0.41], d' = 0.157; Fig. 3*D*).

As expected, both in the threat and control group the greater the proportion of TVs in a trial the more likely participants were to judge the factory as a TV factory [control: TV factory desirable: β_1_ = 25.55, 95% CI [23.20, 27.90], TV factory undesirable: β_1_ = 24.94 [15.62, 34.26], d' = 0.026 (Fig. 3*C*); threat: TV factory desirable: β_1_ = 27.79, 95% CI [19.17, 36.41], TV factory undesirable: β_1_ = 23.04, 95% CI [17.31,28.78], d' = 0.201 (Fig. 3*D*)].

Note, that the total number of pieces of evidence (televisions + telephones) did not differ when participants reached an undesirable or desirable conclusion (*F*_(1,82.77)_ = 1.39, *p* = 0.24), nor did it differ as a function of perceived threat (*F*_(1,81.95)_ = 1.24, *p* = 0.27), neither was there an interaction between these two factors (*F*_(1,82.77)_ = 1.66, *p* = 0.20). Rather, as shown above, it is the proportion of evidence (which signifies the strength of the evidence) needed to reach a conclusion that differed as a function of perceived threat and valence.

Thus far, our analysis suggests that perceived threat leads to a reduction in the strength of the evidence needed to reach undesirable conclusions, although the cause of the threat (anticipating a negative social situation) had nothing to do with the task at hand. We next sought to identify the precise computational factor affected by perceived threat during evidence accumulation.

### Under threat, the drift rate toward undesirable conclusions is greater

Computationally, there are at least two different ways by which perceived threat can lower the strength of evidence needed to reach undesirable conclusions. First, threat may alter the starting point of the accumulation process. That is, if under perceived threat, participants are a priori more likely to believe they are in an undesirable state relative to controls then weaker evidence will be needed to reach that conclusion. Alternatively, perceived threat can enhance the weight given to each piece of negative evidence relative to control. This again will lead to weaker evidence needed to reach an undesirable conclusion.

To tease apart these possibilities we modeled the responses as a drift-diffusion process (Ratcliff, 1978; Ratcliff and McKoon, 2008; Voss et al., 2013) with the following parameters: (1) *t*_(0)_, amount of non-accumulation/non-decision time; (2) α, distance between decision thresholds; (3) z, starting point of the accumulation process; and (4) v, drift rate is the rate of evidence accumulation (for details, see Materials and Methods). Crucially, in models 1 and 3, the starting point was fixed to 0.5, while in models 2 and 4, we allowed the starting point to vary toward one threshold (thus allowing a starting point bias). In models 3 and 4, we allowed the drift rate to vary (which we call a drift rate bias) depending on whether the participant was visiting a desirable factory or an undesirable factory (thus allowing a process bias).

**Figure 4.**
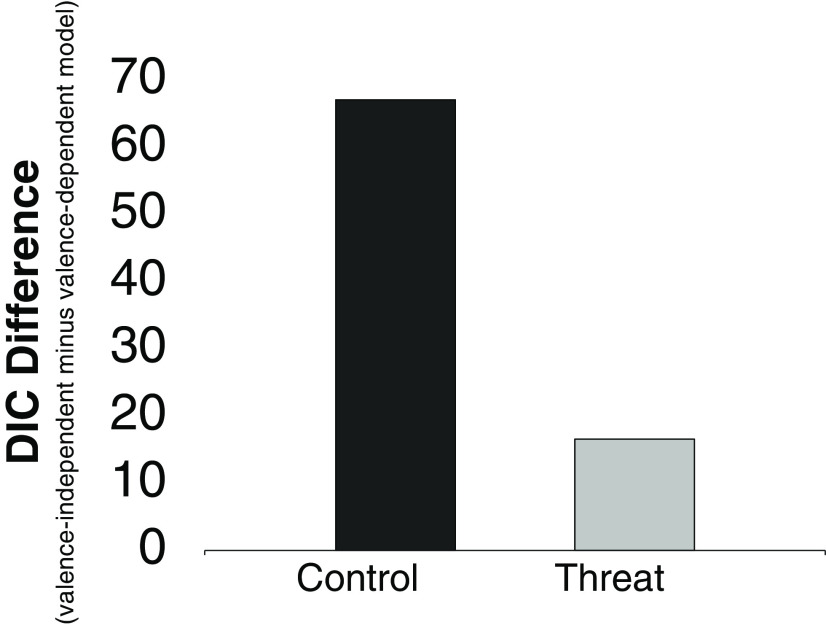
Difference in fit between winning valence-dependent model and valence-independent model as a function of perceived threat. The *y*-axis shows the difference in DIC scores between the valence-independent model and the winning valence-dependent models for the control group (dark gray) and threat group (light gray).

The DIC, a generalization of the AIC for hierarchical models, was calculated for each model (Table 2). The DIC scores indicated that model 4 (the valence-dependent model), which included a valence-dependent starting point and drift rate, outperformed all other models for both threat and control groups. As can be observed in Figure 4, while for the control group the valence-dependent model was clearly a better fit than the valence-independent model (replicating our previous results Gesiarz et al., 2019), for the threat group the advantage in terms of fit was modest.

**Table 2.** Drift diffusion model specification

Number	Model	Starting point (z)	Drift rate (5)	DIC (control)	DIC (threat)
1	Valence independent	z = 0.5	v	11,373.43	7761.38
2	Valence-dependent starting point	0<z < 1	v	11,343.42	7757.88
3	Valence-dependent drift rate	z = 0.5	v = β0 + β1 factory desirability	11,322.83	7758.58
4	Valence-dependent drift rate and starting point	0 < z < 1	v = β0 + β1 factory desirability	11,306.45	7744.82

For each group, we ran four models which differed in whether we allowed the starting point to vary (models 2 and 4), whether we included a valence-dependent drift rate bias (models 3 and 4), or neither (model 1). DIC scores show goodness of fit, with lower numbers indicating better fit.

**Figure 5.**
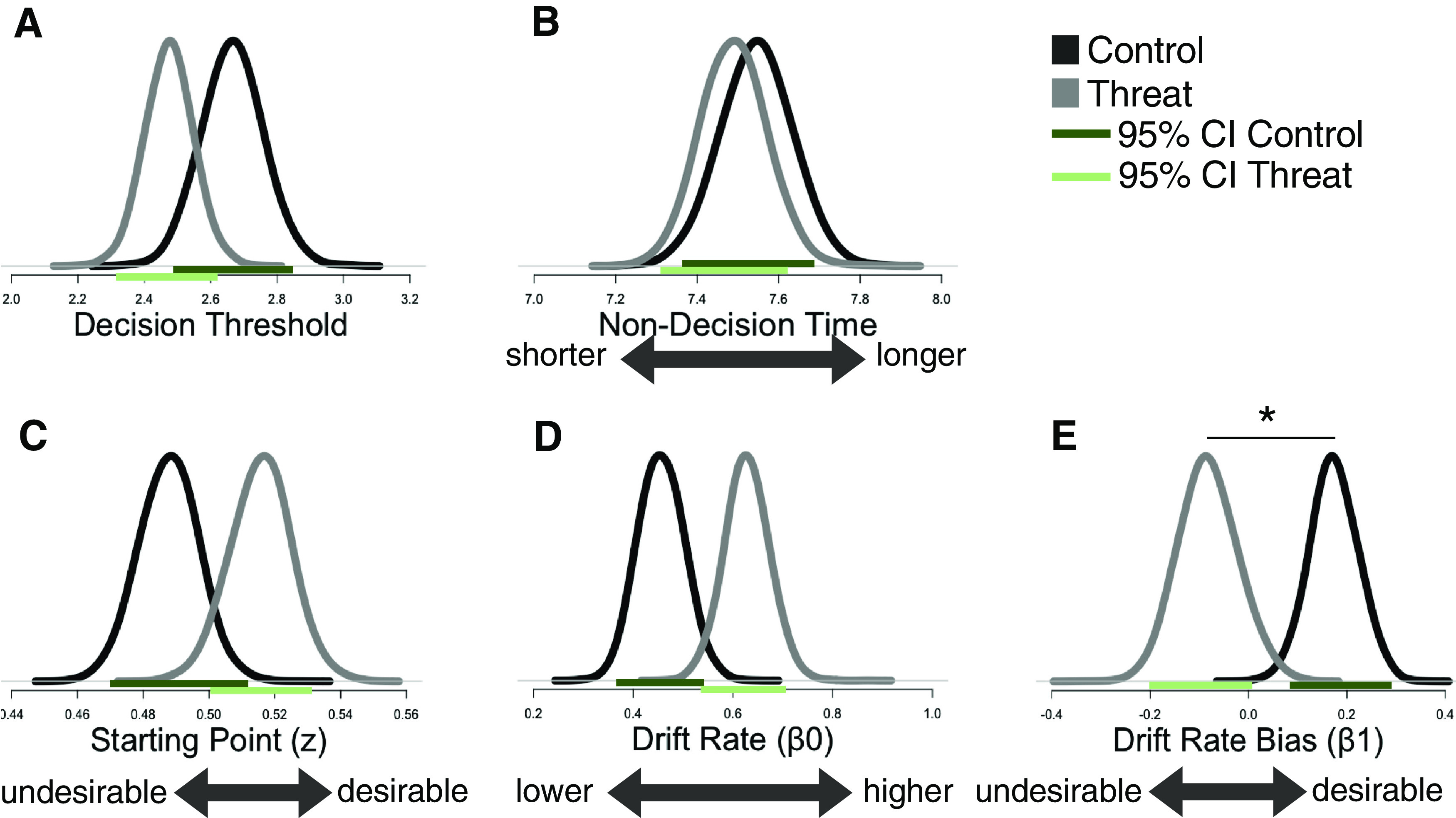
Under threat, valence-dependent drift rate bias is abolished. Displayed are the posterior distributions of parameter estimates for the threat group (light gray) and the control group (black). Light green line indicates 95% CI for the threat group. Dark green line indicates 95% CI for the control group. No significant difference is observed between groups for estimates of (***A***) decision threshold, (***B***) non-decision time, (***C***) starting point, (***D***) drift-rate constant, (***E***) In contrast, a significant difference is observed for the valence-dependent drift rate bias. In the control group, the bias indicates a significantly larger drift rate toward the desirable than undesirable conclusion. This bias is corrected for under perceived threat and is numerically inverse (that is the non-significant bias is negative under perceived threat but significantly positive for controls); * indicates significant difference between parameters in threat and control group (i.e., confidence intervals do not overlap).

We next examined which of the accumulation parameters were affected by perceived threat. As observed in Table 3 and Figure 5, only one element in the accumulation process was significantly altered by perceived threat: the valence-dependent drift rate bias. The drift rate bias is the difference in drift rates between desirable and undesirable factories, the greater the bias the greater the drift rate for desirable factories relative to undesirable ones. As can be observed in Figure 5*E*, the valence-dependent bias in drift rate in the control group was significantly greater than in the threat group (control: β1 = 0.17 [0.07, 0.27]; threat: β1 = −0.08 [−0.20, 0.04]). For controls the bias in drift was significantly *positive* (95% confidence intervals (CI) do not include zero: β1 = 0.17 [0.07, 0.27]), leading to a drift rate that was much higher when participants were in the desirable factory (v_desirable_ = 0.63) than undesirable factory (v_undesirable_ = 0.46). In contrast, under perceived threat, the bias in drift rate was numerically negative and not significantly different from zero (95% confidence intervals (CI) include zero: β1 = −0.08 [−0.20, 0.04]), leading to a drift rate that was numerically and non-significantly larger when participants were in the undesirable factory (v_undesirable_ = 0.63) than desirable factory (v_desirable_ = 0.55).

**Table 3.** Parameter estimates of the evidence accumulation process

Estimate (from data)	Control	Threat
Decision threshold (α)	2.67 [2.49,2.85]	2.47 [2.33, 2.62]
Non-decision time (*t*_(0)_)	7.55 [7.37, 7.71]	7.49 [7.33, 7.64]
Starting point (z)	0.48 [0.47, 0.51]	0.51 [0.50, 0.53]
Intertrial starting point parameter (sz)	0.18 [0.07, 0.27]	0.19 [0.06, 0.28]
Drift rate (β0)	0.46 [0.37, 0.55]	0.63 [0.54, 0.72]
Drift rate bias (β1)	0.17 [0.08, 0.27]	−0.08 [−0.20, 0.04]

Displayed are the model estimates from the winning model for the control and threat groups. These include decision threshold (α), non-decision time (*t*_(0)_), starting point (0 < z < 1), intertrial starting point parameter (sz), constant drift rate (β0), and drift rate bias (β1). The latter is the term reflecting the additional weight added to the drift rate as a function of factory desirability. Positive values indicate a bias toward desirable judgements, and negative values indicate a bias toward undesirable judgements. Confidence intervals in square brackets.

**Figure 6.**
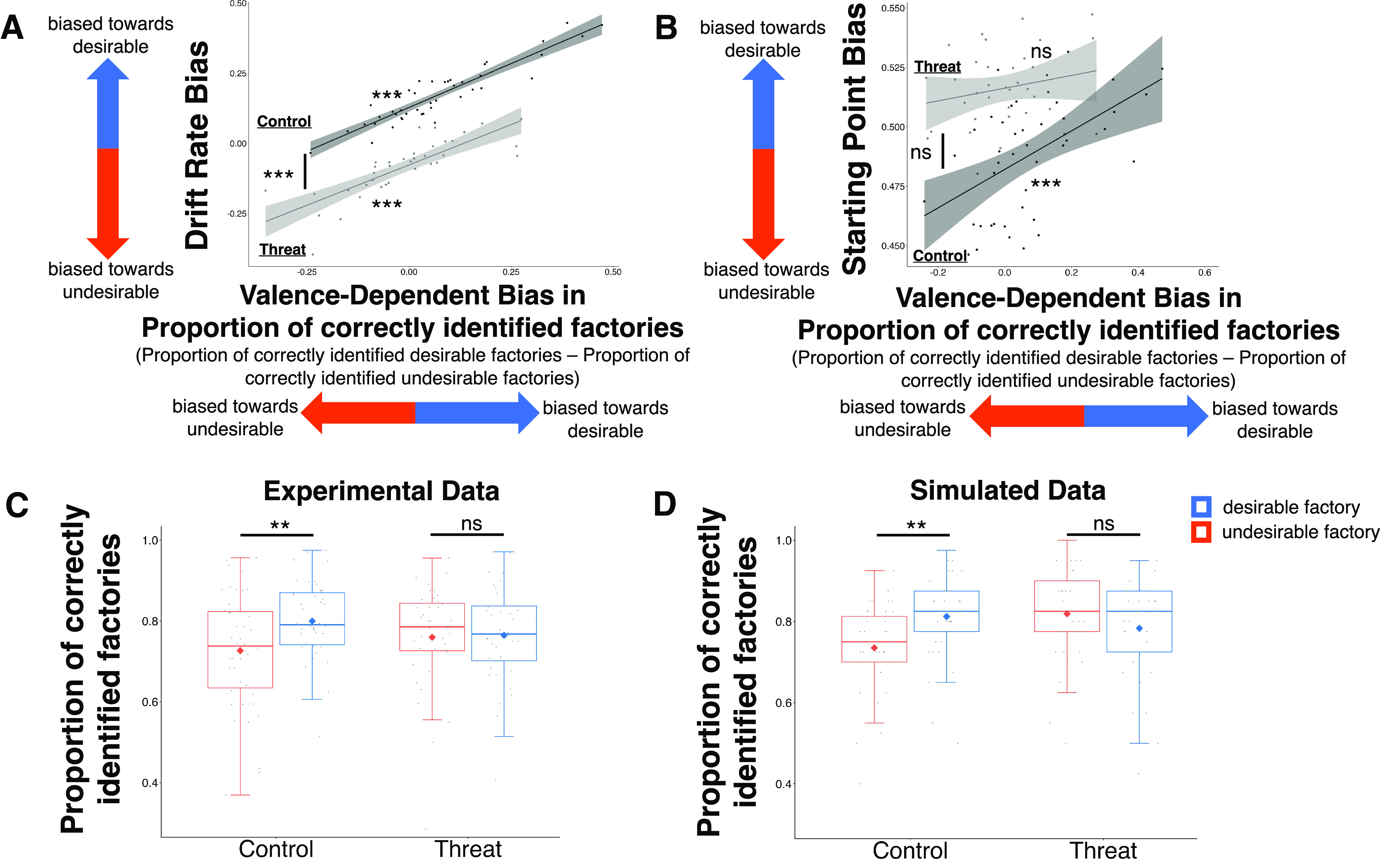
Threat-induced change in valence-dependent drift rate bias is expressed as valence-dependent changes in proportion of correctly identified factories. ***A***, A positive relationship is observed between valence-dependent drift rate bias (*y*-axis) and valence-dependent bias in proportion of correctly identified factories (*x*-axis). Individuals with greater drift rate toward desirable than undesirable conclusions are more likely to correctly categorize desirable than undesirable factories. This is true both for controls (dark gray) and participants under perceived threat (light gray). For controls the regression line is above that of the participants in the threat group, due to their drift rate bias being significantly greater. The regression line for controls is also shifted to the right which indicates significantly greater valence-dependent judgment bias. ***B***, By contrast, we did not observe a relationship between starting point bias (*y*-axis) and valence-dependent bias in proportion of correctly identified factories (*x*-axis) in the threat group (light gray). A positive correlation was observed in the control group (dark gray). In the control group, individuals with a large starting point bias were more likely to correctly identify desirable than undesirable factories. Although the line for the threat group is above that of the control group, this is not a significant difference. ***C***, Controls are less likely to correctly categorize undesirable factories (orange) than desirable factories (blue), while this is not the case for participants in the threat group. ***D***, Simulated data based on model parameters reproduced these findings. Data are plotted as box plots for each condition, in which horizontal lines indicate median values, boxes indicate 25/75% interquartile range and whiskers indicate 1.5 × interquartile range. Diamond shape indicates the mean; **p < 0.01, ns = not significant. Clouds represent CIs.

We simulated data using group parameters from the threat and control group separately (for details, see Materials and Methods). We first examined whether the model parameters could be successfully recovered based on the simulated data. To do so the valence-dependent model was fit to simulated data, in the same way as for the experimental data. We sampled 2000 times from the posteriors, discarding the first 500 as burn in. As shown in Table 4 model parameters could be successfully recovered based on the simulated data. Additionally, we examined whether that the simulated data reproduced the same behavioral pattern of results as the participants' data. This was indeed the case (see Fig. 6*D*; detailed explanation below).

**Table 4.** Recovered parameter estimates of the evidence accumulation process based on simulated data

Estimate (recovered from simulation)	Control	Threat
Decision threshold (α)	2.67 [2.63, 2.72]	2.48 [2.43, 2.53]
Non-decision time (*t*_(0)_)	7.55 [7.51, 7.59]	7.42 [7.39, 7.46]
Starting point (z)	0.51 [0.48, 0.53]	0.51 [0.48, 0.54]
Intertrial starting point parameter (sz)	0.33 [0.20, 0.43]	0.15 [0.01, 0.31]
Drift rate (β0)	0.44 [0.38, 0.51]	0.68 [0.6, 0.77]
Drift rate bias (β1)	0.15 [0.08, 0.22]	−0.14 [−0.22, −0.049]

Displayed are the winning model estimates recovered from simulated data for the control and threat groups. These include decision threshold (α), non-decision time (*t*_(0)_), starting point (0 < z < 1), intertrial starting point parameter (sz), constant drift rate (β0), and drift rate bias (β1). The latter is the term reflecting the additional weight added to the drift rate as a function of factory desirability. Positive values indicate a bias toward desirable judgements, and negative values indicate a bias toward undesirable judgements. Confidence intervals in square brackets.

As DDM parameters are computed partially based on participants' judgements we expected the model-based valence-dependent drift rate bias to correlate across individuals with a valence-dependent bias in judgements. Indeed, across participants there was a strong positive correlation between valence-dependent drift rate bias and the proportion of correctly identified desirable factories minus the proportion of correctly identified undesirable factories [threat group: *r* = 0.802, *p* < 0.001 (Fig. 6*A*), control: *r* = 0.918, *p* < 0.001 (Fig. 6*A*)], which we term “valence-dependent judgment bias”. Individuals with greater drift rate toward desirable than undesirable judgements were more likely to correctly identify desirable factories as desirable when they observed them than undesirable factories when they observed them. In contrast, starting point bias did not correlate with a valence-dependent bias in judgements in the threat group (*r* = 0.223, *p* = 0.191), but did in the control group (*r* = 0.517, *p* < 0.001). In the latter, a larger starting point bias was related to the proportion of correctly identified desirable factories minus the proportion of correctly identified undesirable factories (Fig. 6*B*).

As we have already shown that participants in the control group had a greater drift rate bias than those under perceived threat, it follows that they will also show greater valence-dependent judgment bias. This is exactly what we found. Entering whether a judgment was correct (coded as 1 for correct response and 0 for incorrect) on every trial into a mixed linear model with valence of factory, group and their interaction as fixed and random effects, as well as fixed and random intercepts revealed a group by valence interaction (*F*_(1,77.46)_ = 4.67, *p* = 0.03; Fig. 6*C*) as well as a main effect of factory valence (*F*_(1,77.46)_ = 5.96, *p* = 0.02) and no main effect of group (*F*_(1,80.77)_ = 0.02, *p* = 0.90). To tease apart the interaction we ran the same linear mixed models as above separately for each group. This revealed an effect of valence in the control group (*F*_(1,77.87)_ = 11.06, *p* = 0.001), where participants were less likely to correctly categorize undesirable factories (proportion of correctly categorized factories undesirable factories M = 0.72%, 95% CI [0.68, 0.76]) than desirable factories (proportion of correctly categorized factories desirable factories: M = 0.80%, 95% CI [0.76, 0.84]). In contrast, under perceived threat, the effect of valence disappeared (*F*_(1,80.77)_ = 0.02, *p* = 0.90), participants were as likely to correctly categorize undesirable factories (M = 0.77%, 95% CI [0.73,0.80]) as they were desirable factories (M = 0.76%, 95% CI [0.72, 0.80]). This suggests that under perceived threat, the valence-dependent judgment bias is abolished.

We conducted the same analysis on our simulated data and find that it nicely reproduced the behavioral pattern of results (Fig. 6*D*).

## Discussion

The findings show that perceived threat has a profound effect on the process by which evidence is accumulated. In particular, it leads to a reduction in the strength of the evidence needed to reach undesirable conclusions. Relative to controls, participants under perceived threat required a smaller proportion of negative stimuli to be observed before reaching an undesirable judgment. In contrast, there was no difference between the groups in the strength of evidence accumulated before reaching a desirable judgment. We found this to be true despite the fact that the cause of the threat (anticipating a socially stressful event) was unrelated to the task performed (judging whether more phones or more TVs were observed).

Computationally, there are different mechanisms by which perceived threat can lower the strength of evidence needed to reach undesirable judgements. First, under threat, participants may be a priori more likely to believe they are in an undesirable state relative to controls leading to weaker evidence needed to reach that conclusion. Another possibility is that perceived threat can selectively increase the rate of negative information accumulation (drift rate) relative to control. This again will lead to weaker evidence required to reach an undesirable judgment. To tease apart these possibilities we modeled the responses as a drift-diffusion process (Ratcliff, 1978; Ratcliff and McKoon, 2008; Voss et al., 2013). We found support for the latter. Specifically, perceived threat altered only one feature of the accumulation process: the relative drift rate toward desirable and undesirable judgments (the “valence-dependent drift rate bias”). For controls the bias in drift rate was significantly positive, the rate of information accumulation was greater toward desirable than undesirable conclusions (as observed before Gesiarz et al., 2019). Under threat, however, the bias disappeared because of the drift rate toward undesirable judgment increasing.

The results fit with previous suggestions that perceived threat directs attention toward negative stimuli (Riaz et al., 2017) and leads to greater impact of such stimuli on belief updating (Garrett et al., 2018). Indeed, it is possible that the effect of perceived threat on the rate of negative information accumulation is partially because of increased attention toward negative stimuli. The current findings go beyond these previous demonstrations to illuminate the effects of perceived threat on the process of sequential accumulation and show that weaker evidence is needed to reach undesirable conclusions under threat.

Here, we show a causal link between perceived threat and evidence accumulation in healthy individuals. It is interesting, however, to consider how the findings may be related to evidence accumulation in individuals with affective disorders, as these are often triggered by stressful events and/or characterized by high anxiety. With regards to individuals with high trait anxiety, a processing advantage for threatening words has been previously reported (White et al., 2010). While that study was correlational and thus could not determine whether anxiety caused the changes to drift rate and/or vice versa, our results support the notion that anxiety can in fact alter the drift rate toward undesirable conclusions, even if the anxiety is short lived rather than chronic. With regards to individuals with anxiety and mood disorders, one study (Aylward et al., 2020) found a lower drift rate toward desirable conclusions compared with healthy individuals. Interestingly, the latter study did not detect any effects of induced threat, which may be because of the fact that the task used in that study (as well as in all the above-mentioned studies) unlike ours, was a non-sequential perceptual decision-making task. The process by which pieces of evidence are accumulated over time may be especially impacted by perceived threat.

Our study suggests that evidence accumulation is a flexible process which quickly adjusts to the environment. In particular, the findings show that perceived threat leads to a valence-dependent change to the accumulation process, even when information is not directly related to the cause of the threat. An increased rate of negative information accumulation can then enhance the probability of taking precautionary action to avoid aversive consequences. As aversive outcomes can be more severe and frequent in threatening environments, such generalization can be, on average, adaptive. However, in individuals who are hypersensitive to threat and/or falsely perceive situations as threatening, such as those suffering from anxiety and depression, an increased rate of negative information accumulation could be maladaptive. This is because such increased rate can produce overly pessimistic predictions, which induce stress and anxiety further elevating symptoms.
